# Study on the Effect of Interlayer Tailings Content on the Shear Properties of Geotextile Interface

**DOI:** 10.3390/ma19173620

**Published:** 2026-08-26

**Authors:** Yihan Li, Sheng Liu, Yuan Wang

**Affiliations:** 1 College of Water Conservancy and Hydropower Engineering, Hohai University, Nanjing 210098, China; 231302020068@hhu.edu.cn (Y.L.); wangyuan@hhu.edu.cn (Y.W.); 2 School of Civil and Safety Engineering, Wanjiang University of Technology, Maanshan 243031, China

**Keywords:** geotextile, tailings content between geotextile layers, interface shear properties, tailings dam, stability

## Abstract

To investigate the influence of tailings content between geotextile layers in tailings dams built with geotextile bags on interfacial shear behavior and dam stability, direct shear tests were conducted to examine the shear mechanical properties of the geotextile interfaces under different tailings water contents (20%, 30%) and different tailings contents per unit area between geotextile layers (0, 0.0125, 0.0250, 0.0500 g/cm^2^). Based on the distribution patterns of tailings particles at the shear plane, the interface shear mechanism was elucidated, and the influence of interlayer tailings content on the stability of the tailings dam built with geotextile bags was analyzed using the discontinuous–continuous coupling method. The results show that, under the tested material properties and experimental conditions, both the peak shear stress and the interfacial friction angle first increased and then decreased with increasing tailings content between geotextile layers, reaching their maximum values when the tailings content was 0.0250 g/cm^2^, while the cohesion exhibited the opposite trend. An appropriate amount of tailings particles embedded in the pores of the geotextile enhances the mechanical interlock between the particles and the geotextile, thereby improving the shear resistance at the interface. When the tailings content was excessively high, a loose tailings layer formed at the interface, shifting the shear plane into the tailings and, consequently, reducing the interface shear strength. Under the tested conditions, the safety factor of the dam first increased and then decreased as the tailings content between geotextile layers increased, reaching its highest value at a tailings content of 0.0250 g/cm^2^, which was approximately 14.3% higher than under conditions without tailings. When the tailings content increased to 0.0500 g/cm^2^, the safety factor of the dam decreased. This research provides a theoretical basis for quality control in the construction of tailings dams built with geotextile bags and the design of geotextile interfaces under conditions comparable to those investigated in this study.

## 1. Introduction

**Figure 1:**
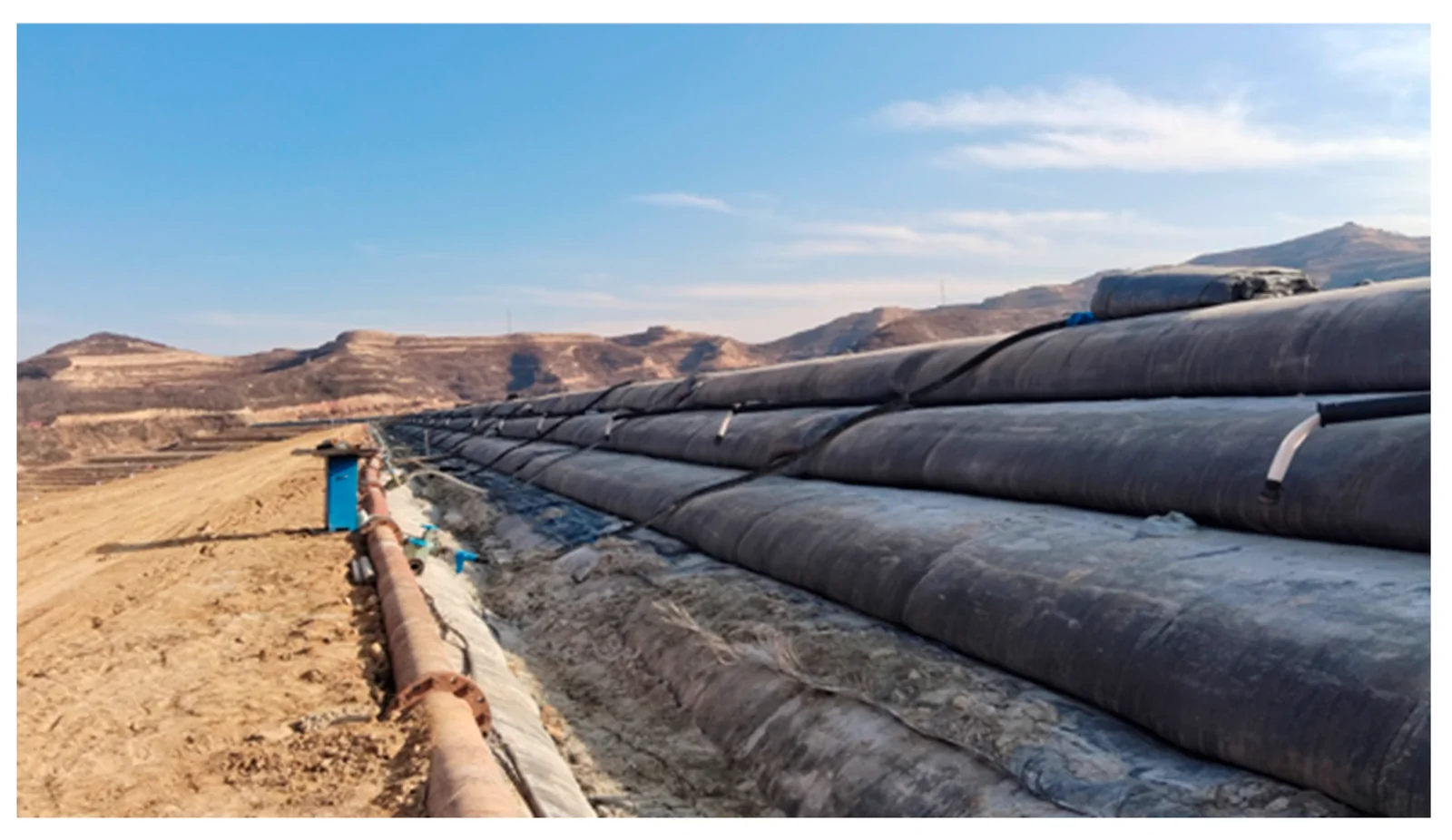
The tailings dam built with geotextile.

With advances in mineral processing and mineral recovery technologies, the particle size of tailings stored in tailings reservoirs has become progressively finer [1,2]. The traditional upstream method for constructing dams for fine-grained tailings is generally associated with engineering challenges such as slow tailings consolidation, difficulties in dam construction, and poor dam permeability [3,4,5,6], which compromise the safe operation of the dam. Tailings dams built with geotextile bags (as shown in Figure 1), as a new type of embankment technology, possess advantages such as high dam strength and rapid construction, holding broad application prospects in the remediation and reinforcement of fine-grained tailings reservoirs [7,8,9,10]. After filling geotextile bags with tailings and allowing drainage and consolidation, a geosynthetic composite with good structural integrity—the geotextile body—forms [11]. The geotextile dam construction method involves stacking multiple geotextile body assemblies in a staggered pattern to form a “dam shell” for the tailings dam, which serves to contain and protect the tailings within the tailings reservoir. Currently, geotextile has been applied in projects such as the Dapingzhang Tailings Reservoir and the Tangdan Tailings Reservoir in Yunnan province of China.

Currently, domestic and foreign scholars have conducted extensive research on geotextile bags [12,13,14,15,16,17,18]. Concerning geotextile bags filled with fine-grained tailings, existing studies have primarily focused on their filling characteristics, consolidation properties [19,20,21], and mechanical properties [22,23,24,25,26]. Li et al. [9,23] examined the friction coefficient at the geotextile interface under different consolidation times through slope slip and direct shear tests. They also investigated the lateral deformation behavior of the geotextile bags under various vertical pressures through unconfined uniaxial compression tests. Silva et al. [22] established a finite element model of geotextile tubes filled with gold mine tailings to investigate their stress–strain behavior under different filling pressures and material properties. Liu et al. [24] studied the interfacial shear strength of tailings–tailings, tailings–geotextile, and geotextile–geotextile under different consolidation times and various filling degrees. Lashkari [25] et al. conducted direct shear tests on three types of tailings with different densities to investigate the instability in the interfaces between iron ore tailings and woven geotextile. Li et al. [26] mixed cement into fine tailings slurry to produce geotextile bags and investigated the effects of cement content on the bags’ permeability and interfacial friction characteristics. Results showed that adding cement to fine tailings slurry can improve the shear strength and residual strength ratio of the interface of geotextile bags.

**Figure 2:**
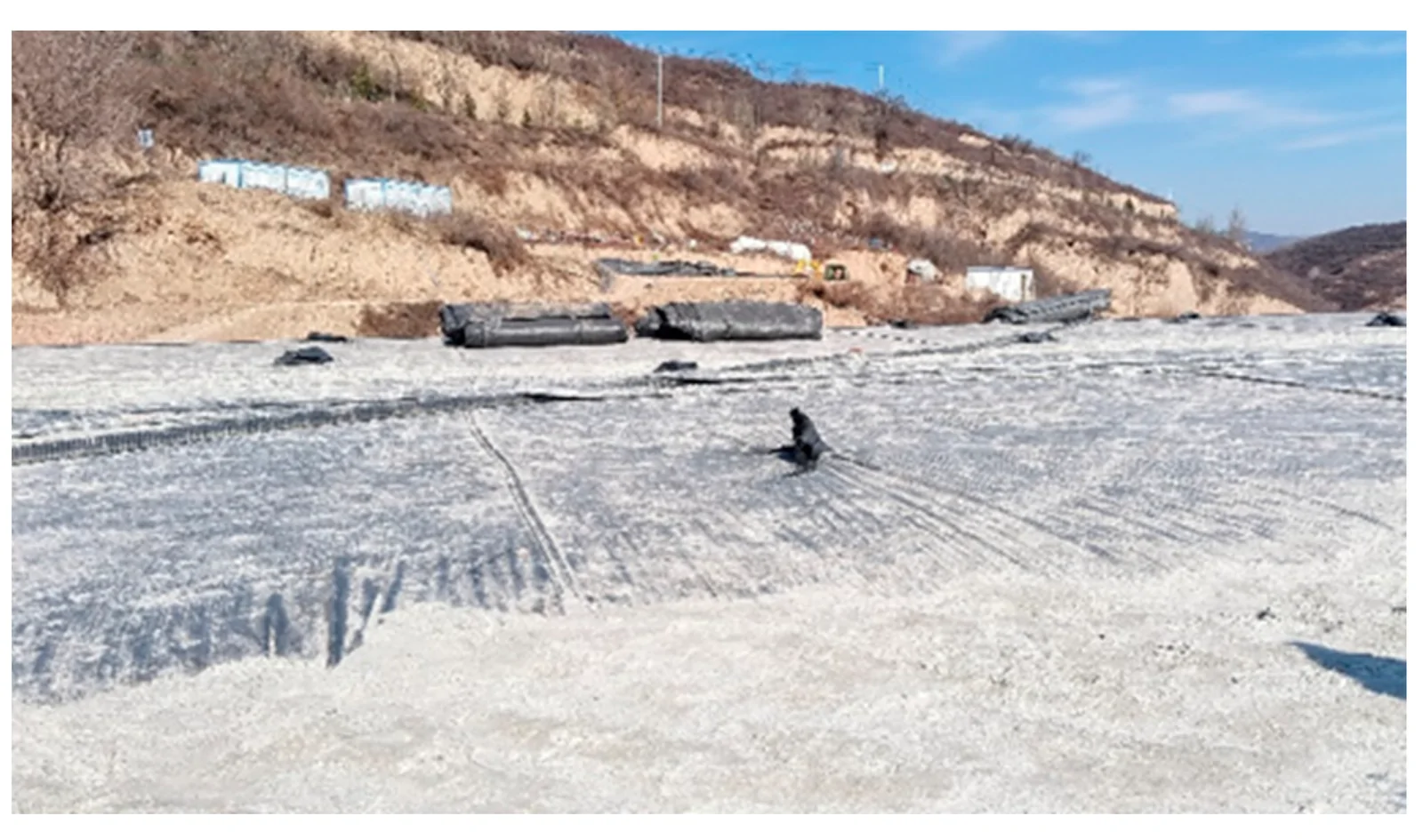
Residual tailings on the surface of the geotextile bags.

Current studies have predominantly focused on the effects of factors such as filling degree, consolidation time, and additive content on the shear properties at the geotextile–tailings interface and the geotextile–geotextile interface. During the construction of tailings dams with geotextile bags, a certain amount of tailings adheres to the surface of the geotextile, as shown in Figure 2, causing the actual contact configuration between adjacent geotextile bags to change from the idealized “geotextile–geotextile” interface to the “geotextile–tailings–geotextile” composite interface. Unlike conventional two-material interfaces, the shear behavior of this composite interface may be governed not only by geotextile–tailings friction but also by the amount of interlayer tailings, which can alter particle–geotextile mechanical interlocking and the location of the shear plane. However, the influence of interlayer tailings content on the shear behavior and failure mechanisms of the geotextile–tailings–geotextile composite interface, as well as its implications for the overall stability of geotextile-bag tailings dams, remains insufficiently understood. This lack of understanding makes it difficult to establish rational criteria for controlling residual tailings during construction and to accurately evaluate the shear resistance of interlayer interfaces in stability analyses.

Furthermore, existing methods for evaluating the stability of tailings dams built with geotextile bags are primarily based on continuum mechanics, considering the entire tailings dam as a single continuum and employing either the classical slice method [9,10,27] or the finite-element-based strength reduction method [28,29] for stability analysis. These computational methods inadequately account for slippage and separation between the sub-dam built with geotextile bags and the tailings in the reservoir.

To explore the mechanism behind the effect of tailings content between geotextile layers on interfacial shear properties and dam stability, this study conducted direct shear tests on the geotextile–tailings–geotextile interfaces under different tailings water content, tailings content between geotextile layers, and normal stress. The study analyzed the influence of tailings content between geotextile layers on peak interfacial shear stress and shear strength parameters, and investigated the mechanism by which interlayer tailings content affects interfacial shear behavior by analyzing the distribution patterns of tailings particles at the interface after shear tests. A numerical model of a tailings dam built with geotextile bags based on the discontinuous–continuous coupling method was established. The interface shear strength parameters obtained from experiments were applied to the stability analysis of the tailings dam built with geotextile bags. The study explored the influence of interlayer tailings content on the dam’s safety factor and the deformation properties of the sub-dam built by geotextile bags, providing a reference for the engineering design and safety analysis of tailings dams built with geotextile bags.

## 2. Experiment Equipment and Scheme

### 2.1. Experimental Materials and Equipment

During the construction process of filling geotextile bags, fine-grained tailings with high water content and small particle sizes prove difficult to retain within the geotextile bags. Therefore, the tailings slurry must be classified using a hydrocyclone, and the portion with larger particle sizes is selected for filling the geotextile bags. The tailings used for this study were obtained from a tailings dam in Gansu Province; their basic parameters are shown in Table 1. The geotextiles are black polypropylene bags from the same construction site, and their basic mechanical parameters are shown in Table 2.

**Figure 3:**
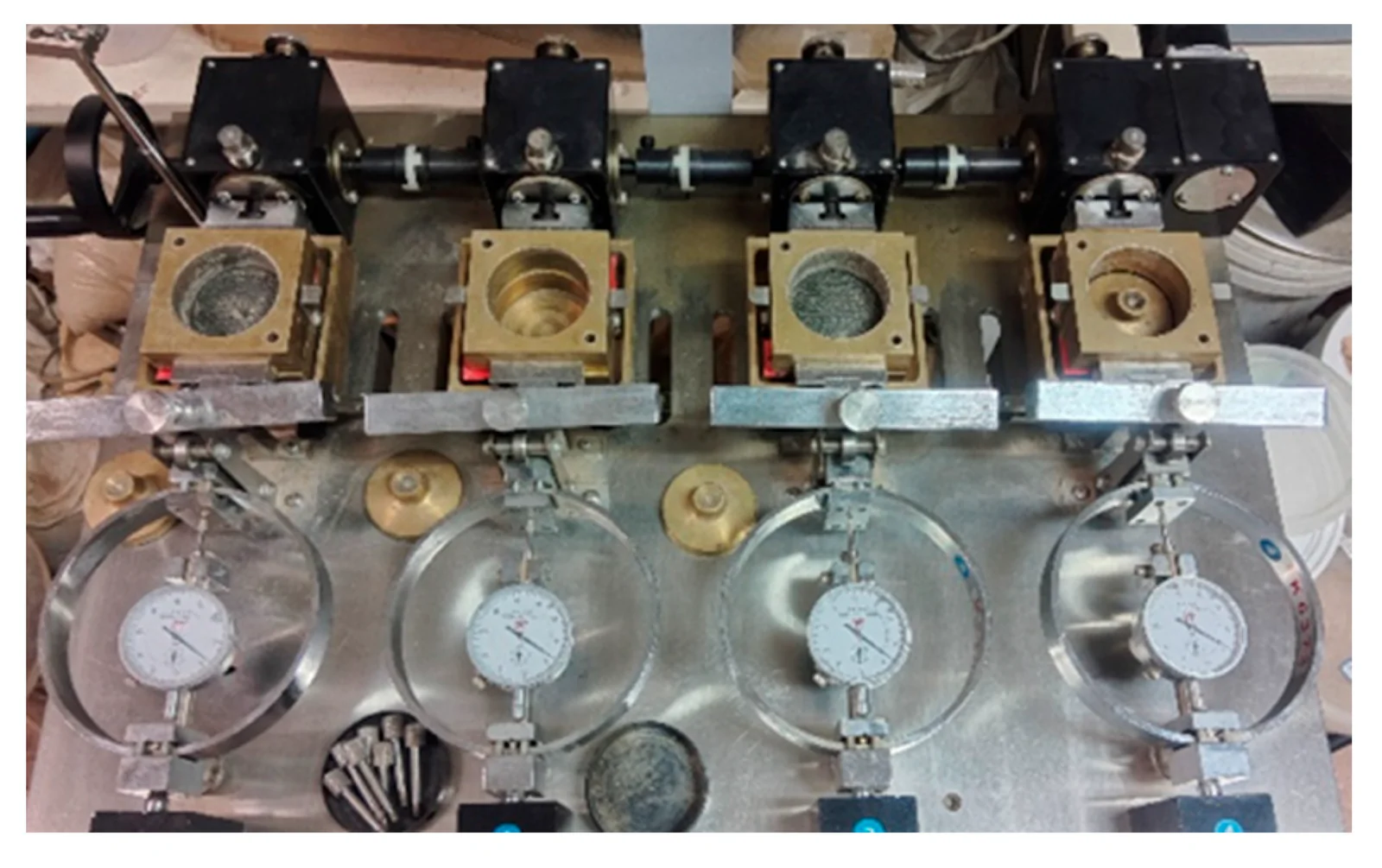
Four-station direct shear device.

In this study, a four-station direct shear device was employed to conduct direct shear tests at the geotextile–tailings–geotextile interface. The device is shown in Figure 3. The main components of this direct shear device include a vertical loading component, a motor for applying horizontal thrust, a horizontal displacement sensor, and upper and lower shear boxes. It is capable of conducting shear tests on four specimens simultaneously under different vertical loads. The shear box can accommodate specimens with a diameter of 61.4 mm and a height of 1 cm.

### 2.2. Experimental Process

To simulate the surface morphology of geotextile in actual engineering conditions, a certain mass of tailings was layered between the geotextile in shear tests, investigating the effect of the tailings content between geotextile layers on the shear properties at the interface. The tailings content between geotextile layers is quantified by the amount of tailings per unit area of geotextile. The experimental conditions were selected based on field investigations, previous studies on the consolidation behavior of tailings-filled geotextile bags, and the actual engineering conditions of the study site. The interlayer tailings content per unit area of the geotextile is set at 0, 0.0125 g/cm^2^, 0.0250 g/cm^2^, and 0.0500 g/cm^2^, respectively; specifically, 0.75 g, 1.50 g, and 3.00 g of tailings are added between each pair of geotextiles, covering the range of tailings accumulation observed at different locations of the geotextile interface in the field. The tailings water content inside the geotextile bags is considered under two conditions: 20% and 30%, representing relatively lower and higher moisture states identified from field measurements and previous consolidation studies [20]. Shear tests of the geotextile–tailings–geotextile interface were conducted under different tailings masses between geotextile layers and different tailings water contents, with the condition of no interlayer tailings serving as the control group. To reduce random experimental errors, each test condition was repeated 3–4 times. For the shear stress–displacement curves and subsequent calculation of the interfacial shear strength parameters, the test result whose peak shear stress was closest to the mean peak shear stress of the repeated tests under the same condition was selected as the representative curve. The detailed test schemes are shown in Table 3.

**Figure 4:**
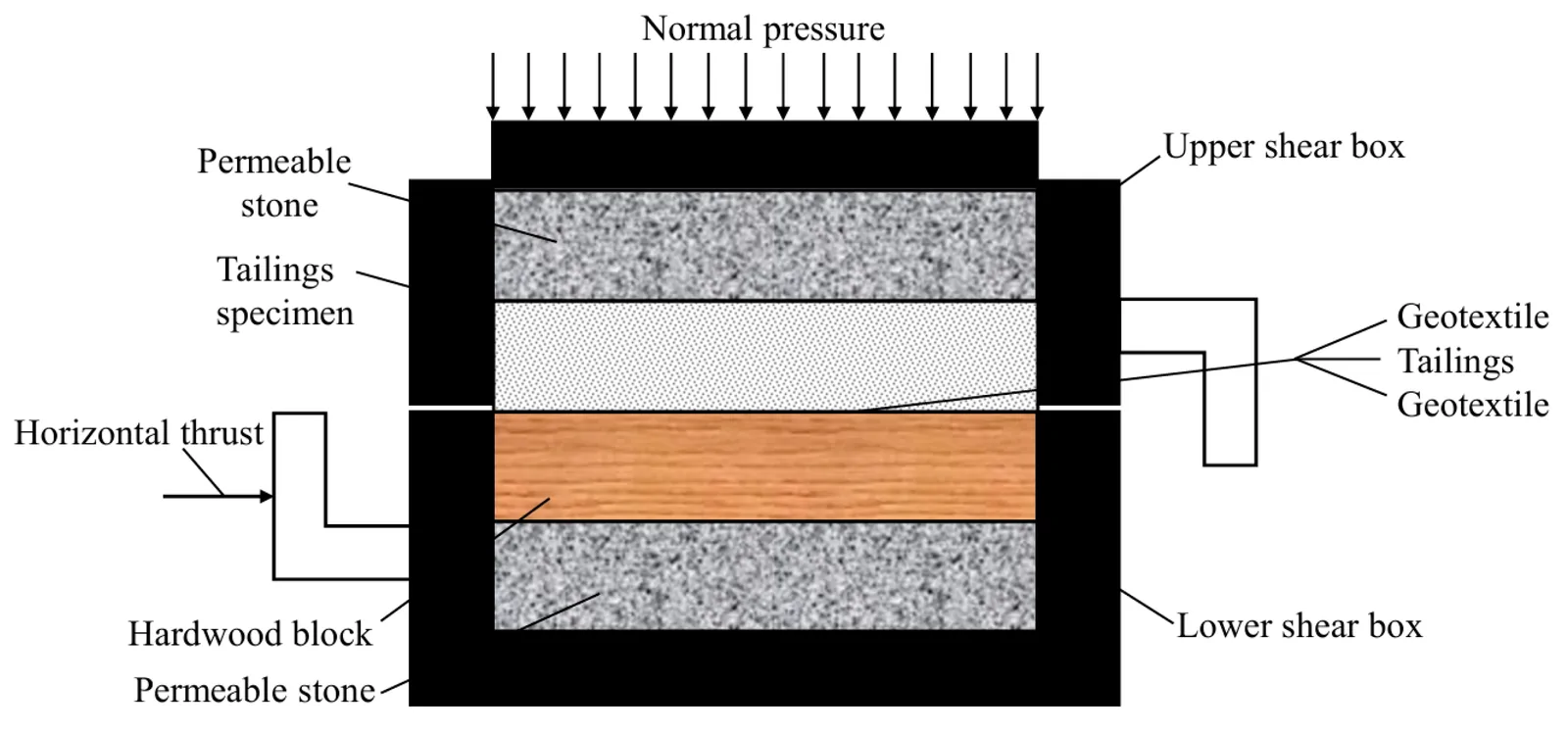
Schematic diagram of the shear box.

**Figure 5:**
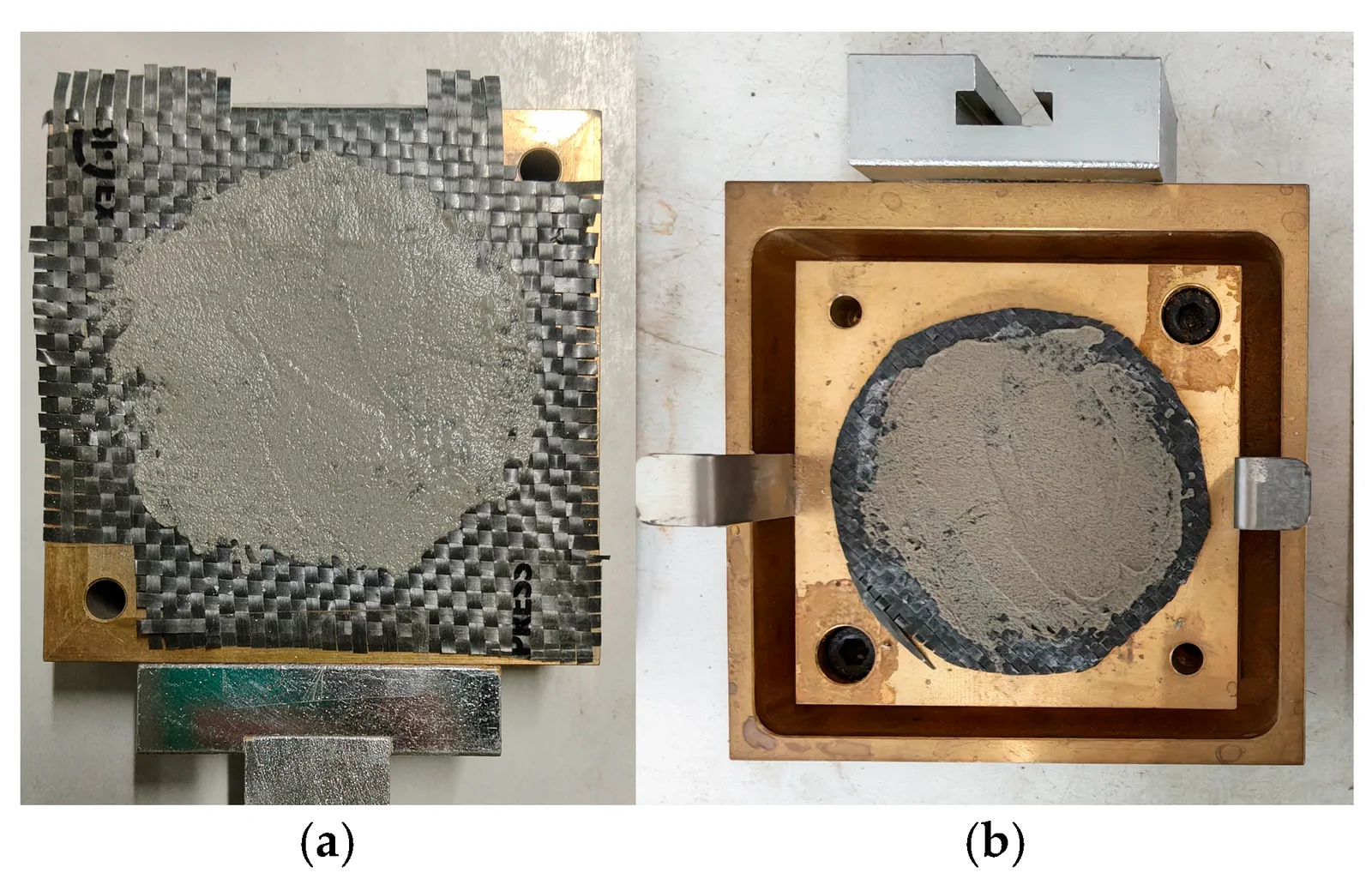
The geotextile–tailings–geotextile interface shear test device. (**a**) Upper shear box. (**b**) Lower shear box.

First, test specimens of tailings with different water contents were prepared. The dry bulk density of the tailings was 1.52 g/cm^3^. A certain mass of dry tailings and water were weighed in the appropriate ratio, and the water was added to the tailings in several batches, followed by thorough mixing. After curing for 24 h, the required mass of wet tailings was poured into a cutting-ring compactor and the tailings specimens were compacted through static pressure. After specimen preparation was complete, a permeable stone was placed at the bottom of the lower shear box, and then the wooden block with the geotextile affixed to its surface was inserted. The geotextile was affixed to the underside of the upper shear box to prevent relative slippage between the geotextile and the upper shear box. The required amount of tailings was divided into two equal portions and a scraper was used to apply it evenly over the geotextile surfaces of the upper and lower shear boxes. Then, the upper shear box and the tailings specimen were installed. After fixing the shear box with screws, the cutting ring with the flat edge facing downward was placed, aligning it with the opening of the shear box, and the filter paper and permeable stones were placed on the top of the tailings specimen. Then, the specimen was slowly pushed into the shear box using the box cover. Figure 4 and Figure 5 show a schematic diagram and a photograph of the shear box for the direct shear test at the geotextile–tailings sand–geotextile interface. When conducting shear tests at the geotextile- geotextile interface, no tailings sand is applied on the surfaces of the upper and lower geotextile bags. The remaining procedure is the same as that for the direct shear test at the geotextile–tailings–geotextile interface.

After specimens were loaded, the shear box was placed on the slide rail of the direct shear device. Normal pressure was applied to the upper shear box, then the screws were removed. And the direct shear test was started. Considering the actual pressure exerted on the geotextile body in the tailings dam, four normal stresses were set at 50 kPa, 100 kPa, 150 kPa, and 200 kPa, respectively. The shear rate was set to 0.8 mm/min, ensuring that the specimen shears within 3 to 5 min. The readings from the horizontal force-measuring device were recorded during the shear process. If the shear stress reading stabilizes or shows a significant decline, stop shearing when the shear deformation reaches 4 mm. If the shear stress reading continues to increase, stop shearing when the shear deformation reaches 6 mm.

## 3. Results and Discussions

### 3.1. The Effect of Water Content and Interlayer Tailings Content on Interface Shear Properties

**Figure 6:**
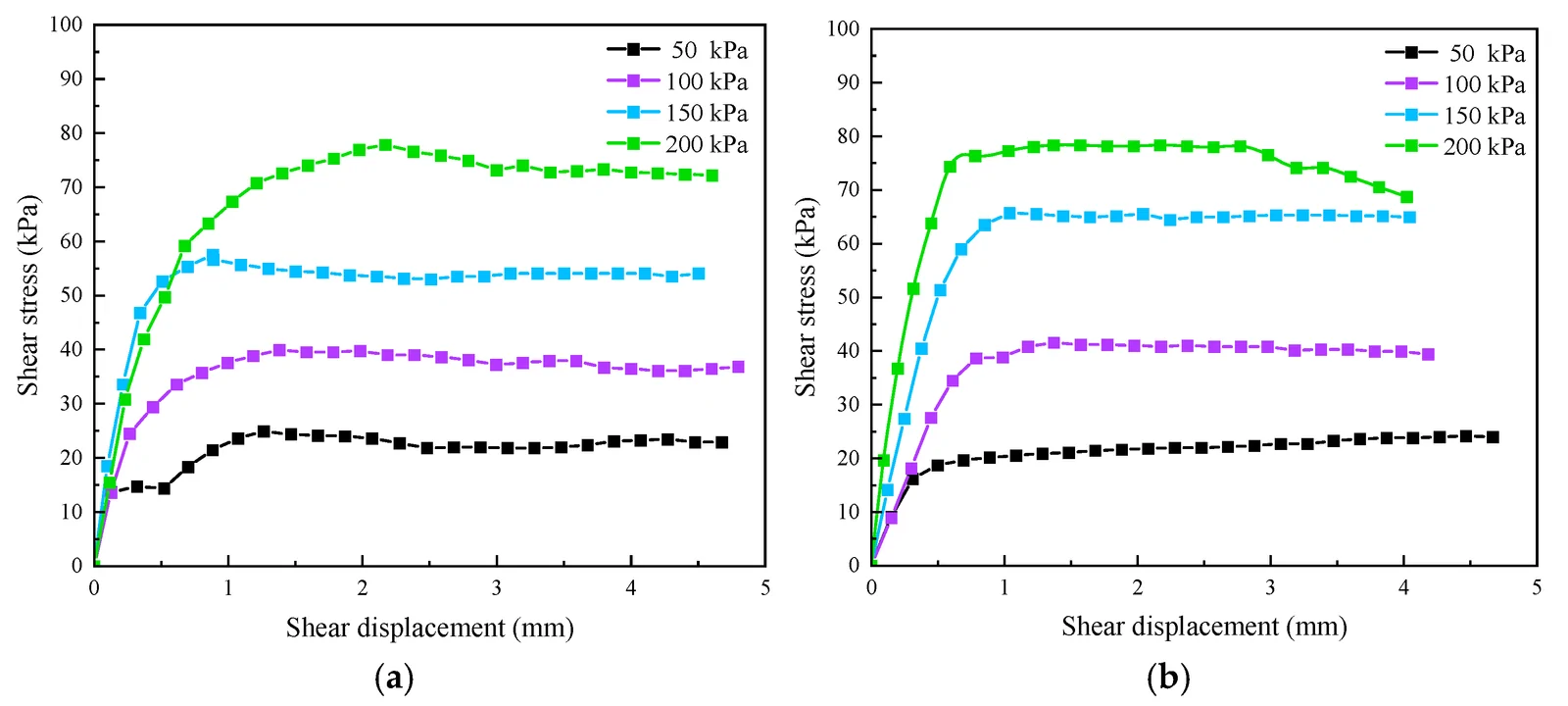

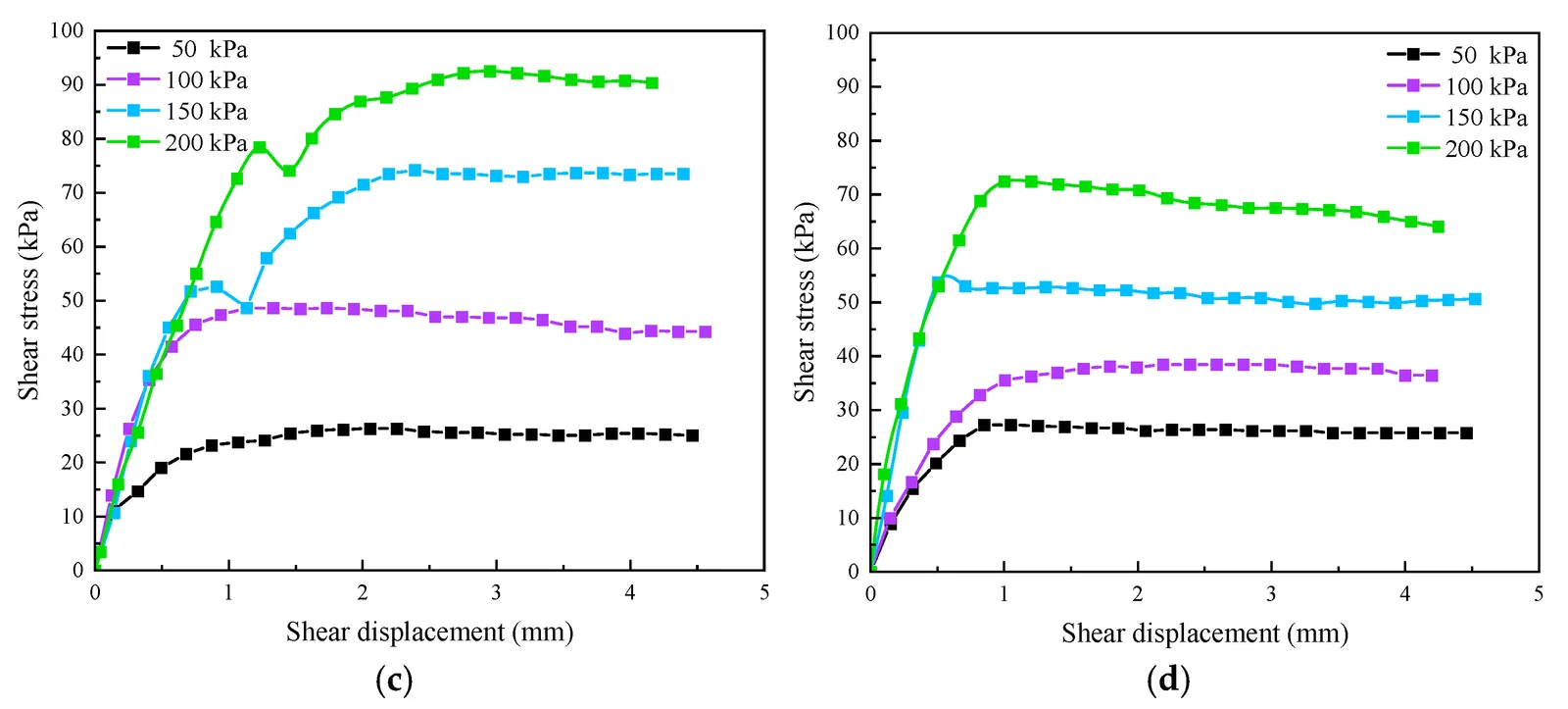
Shear stress and displacement curves of various interfaces under a water content of 20% in tailings. (**a**) Geotextile–geotextile interface. (**b**) Tailings content of 0.0125 g/cm^2^. (**c**) Tailings content of 0.0250 g/cm^2^. (**d**) Tailings content of 0.0500 g/cm^2^.

**Figure 7:**
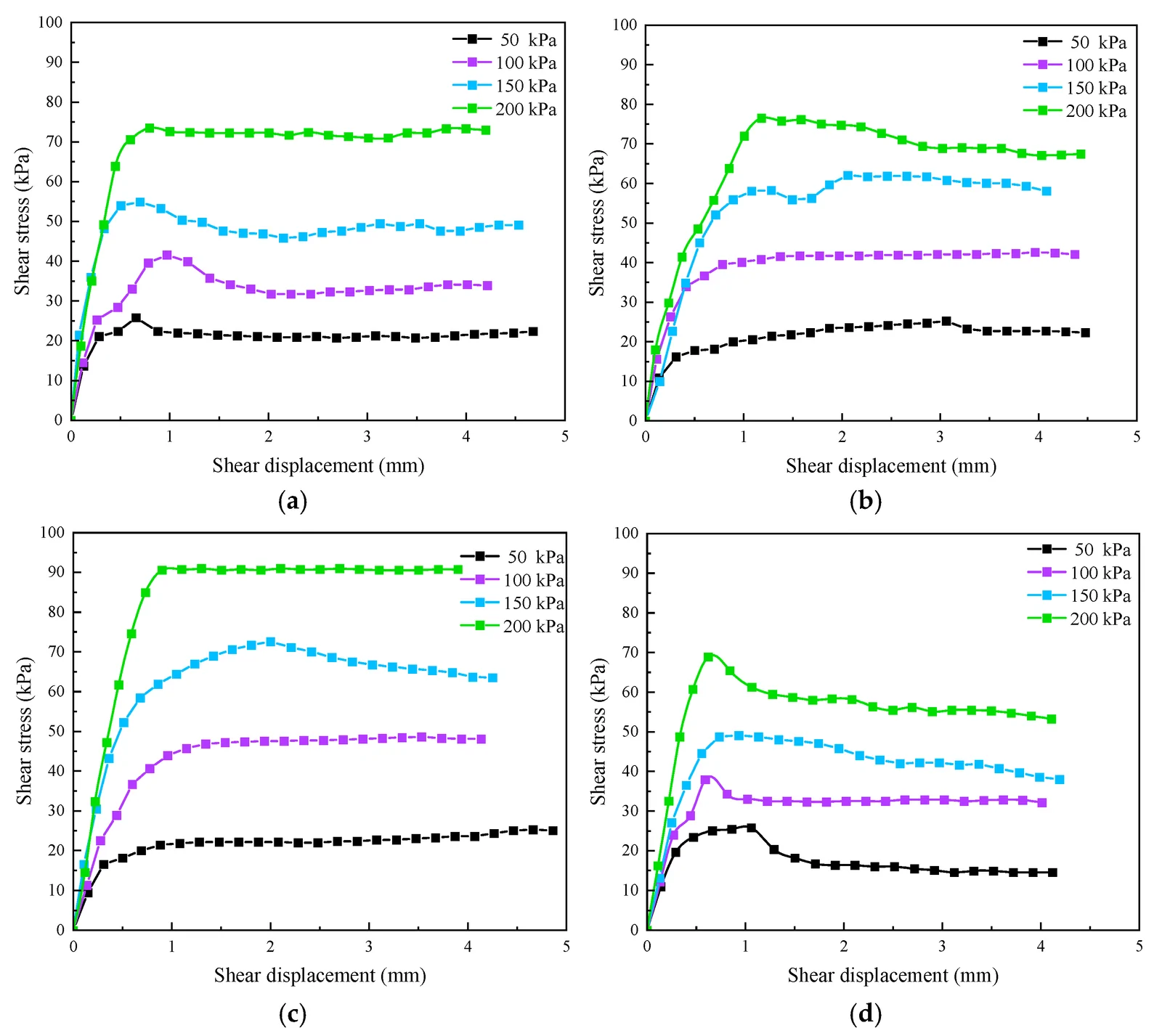
Shear stress and displacement curves of various interfaces under a water content of 30% in tailings. (**a**) Geotextile–geotextile interface. (**b**) Tailings content of 0.0125 g/cm^2^. (**c**) Tailings content of 0.0250 g/cm^2^. (**d**) Tailings content of 0.0500 g/cm^2^.

Figure 6 and Figure 7 show the shear stress–shear displacement curves at the geotextile–tailings–geotextile interface under different tailings content between geotextile layers, with the tailings water content of 20% and 30%. In the initial stage of shearing, the shear stress at the geotextile–tailings–geotextile interface increased with the growth of shear displacement. As the shear displacement continued to increase, the shear stress remained stable or decreased slightly after reaching a peak, exhibiting nonlinear shear hardening behavior.

**Figure 8:**
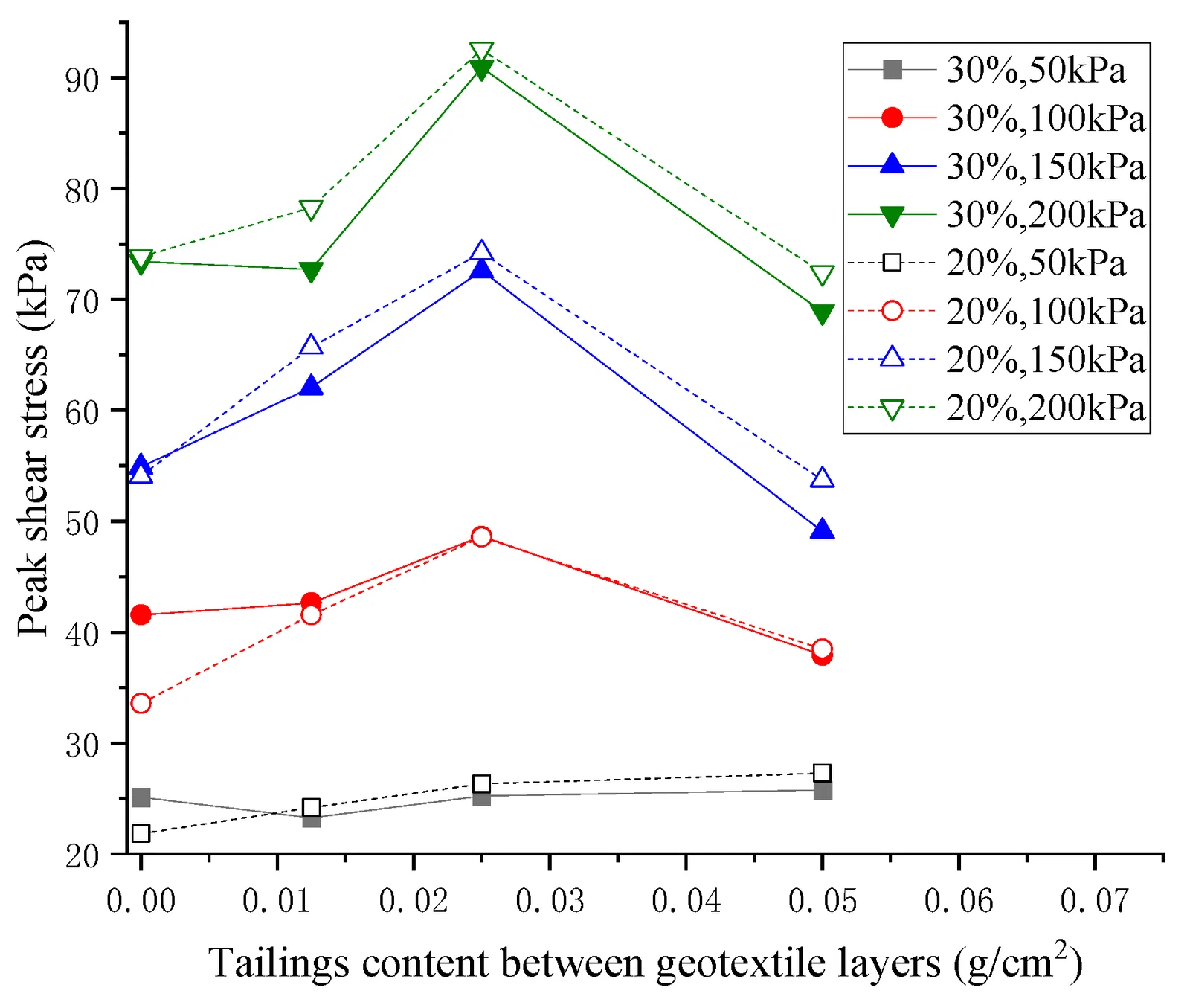
The variation curve of the peak shear stress with the tailings content between geotextile layers.

Figure 8 shows the variation in peak interfacial shear stress with the tailings content between geotextile bags. Overall, under both 20% and 30% water content conditions, the peak shear stress exhibited a trend of first increasing and then decreasing as the tailings content between layers increased. When the tailings content per unit area between geotextile layers is 0.0250 g/cm^2^, the peak shear stress reached its maximum. As the tailings content continued to increase to 0.0500 g/cm^2^, the peak shear stress decreased significantly. When the normal stress was 200 kPa and the water content of the tailings was 20%, the peak interfacial shear stress for a tailings content of 0.0250 g/cm^2^ was 27.8% higher than that for a sand content of 0.0500 g/cm^2^. This is primarily because an appropriate amount of tailings particles embedded at the geotextile interface increased the surface roughness and interlocking effect, thereby effectively improving shear strength. However, when the tailings content was too high, the shear plane shifted into the loose sand layer, causing the strength to decrease. The peak shear stress increased remarkably as the normal stress increased, and the effect of tailings content became more significant under high normal stresses. When the normal stress exceeded 150 kPa, the peak shear stress for a 20% water content was slightly higher than that for a 30% water content under the same normal stress, indicating that a lower water content helps enhance the frictional interlocking effect at the geotextile interface.

**Figure 9:**
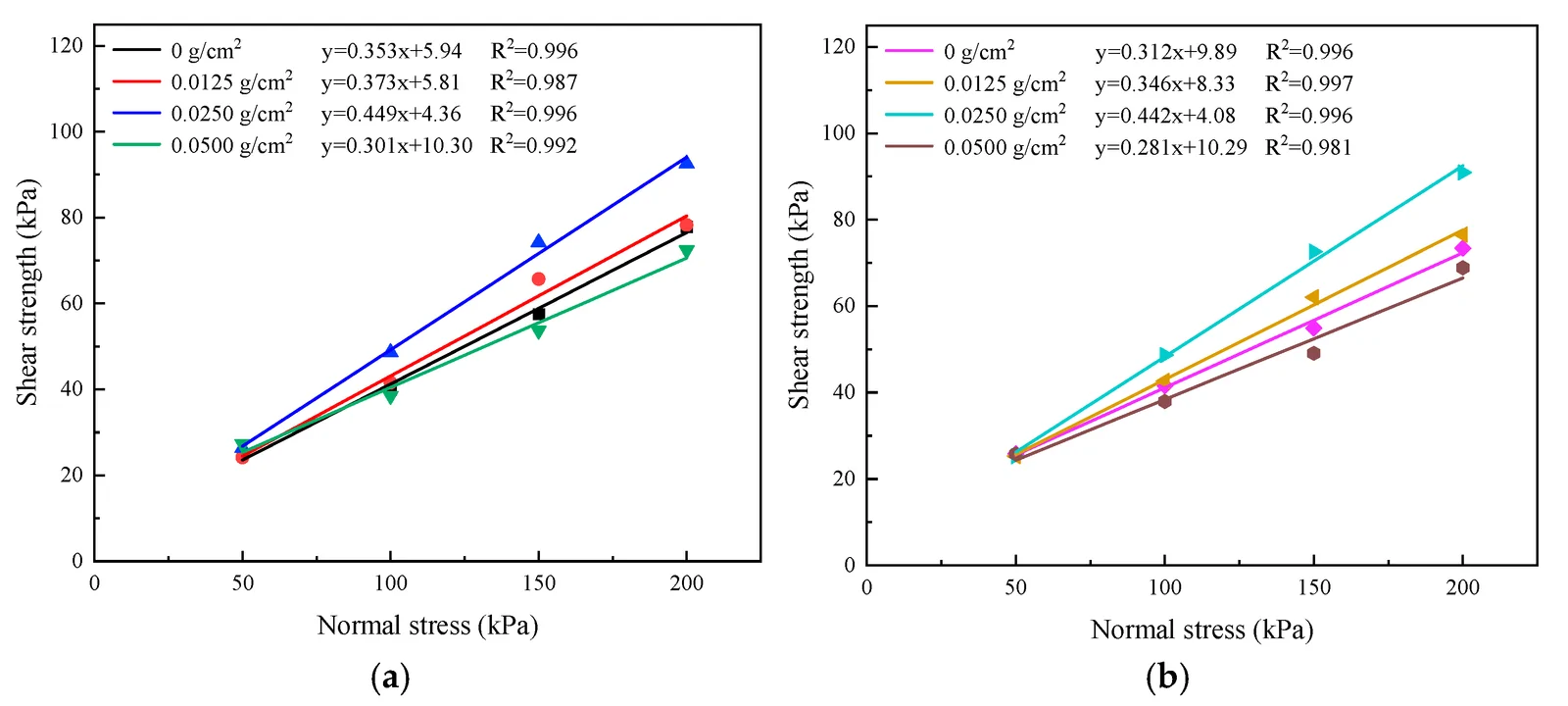
Variation in shear strength at different interfaces with normal stress. (**a**) Water content of tailings is 20%. (**b**) Water content of tailings is 30%.

At the same water content, the shear strength at the geotextile interface increased along with an increase in normal stress. Figure 9 shows the linear fit curves of the shear strength at the geotextile interface versus normal stress under different test conditions. The R-squared values of the fit curves were all larger than 0.98, indicating that a linear function could be used to fit the relationship between the two variables. The fitting results indicate that the shear strength at the geotextile–tailings–geotextile interface followed the Mohr–Coulomb strength criterion, where the slope of the straight line represented the internal friction angle of the interface, and the Y-intercept of the straight line represented the cohesion of the interface.

**Figure 10:**
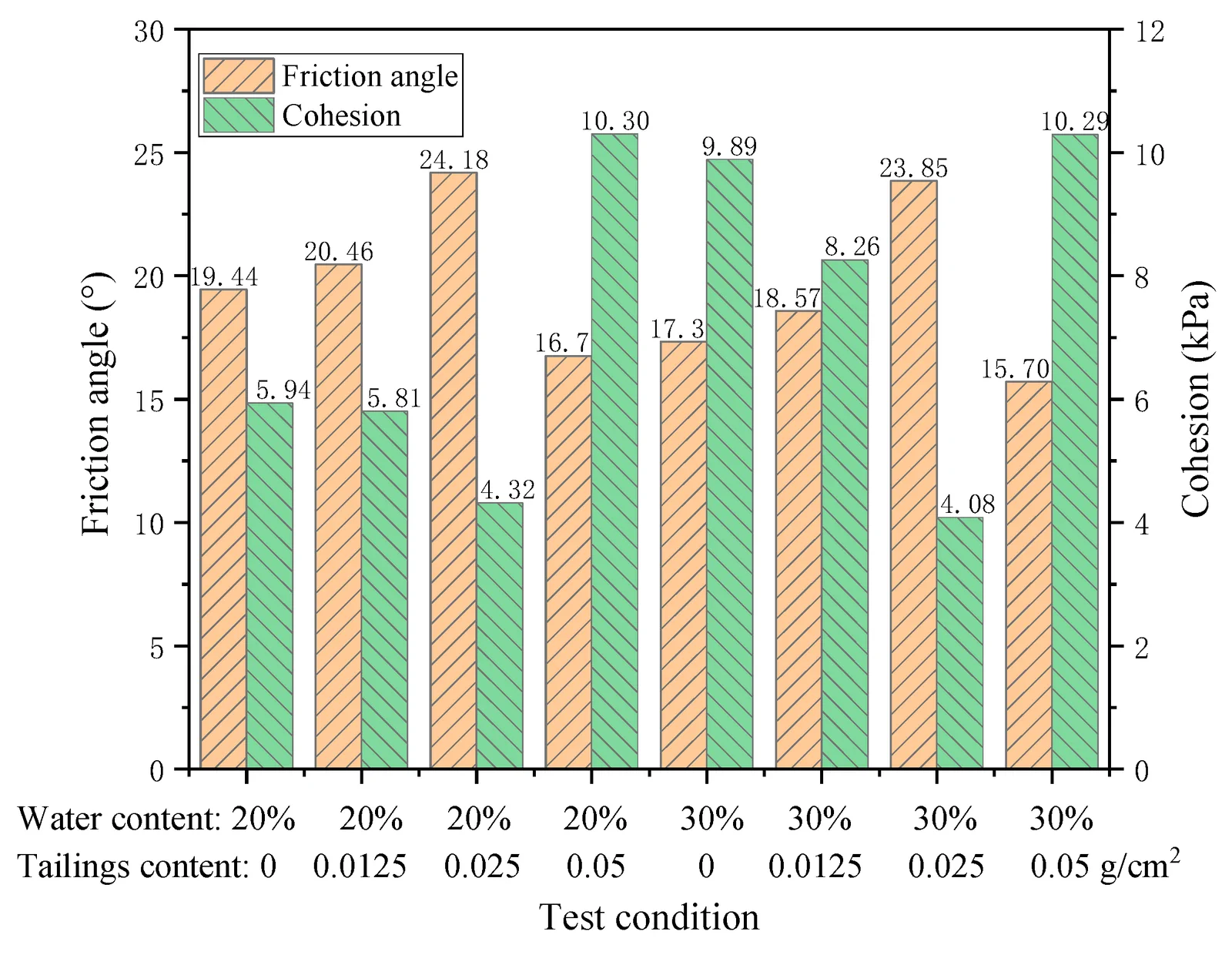
Shear strength parameters of the geotextile interface under different test conditions.

Figure 10 shows the shear strength parameters for various interfaces under different water contents and interlayer tailings contents. The tailings content between geotextile layers had a significant impact on their friction properties. As the interlayer tailings content increased, the friction angle of the geotextile interface first increased and then decreased, while the cohesion first decreased and then increased. When the tailings content per unit area of geotextile was 0.025 g/cm^2^, the friction angle was at its maximum, and the cohesion was at its minimum. As the tailings content continued to increase, the friction angle began to decrease. When the tailings content reached 0.050 g/cm^2^, the friction angle dropped to its minimum value. When the water content of tailings was 20%, the friction angle at a tailings content of 0.050 g/cm^2^ was 13.8% lower than that under conditions where the tailings content between geotextile layers was zero. For the same tailings content per unit area of geotextile, when the water content of tailings was 20%, the friction angle was higher than that when the water content was 30%. An appropriate amount of tailings between geotextile layers can increase the friction angle of the interface to some extent, while an excessive amount of tailings will reduce it.

### 3.2. The Influence Mechanism of Interlayer Tailings Content on Shear Characteristics

**Figure 11:**
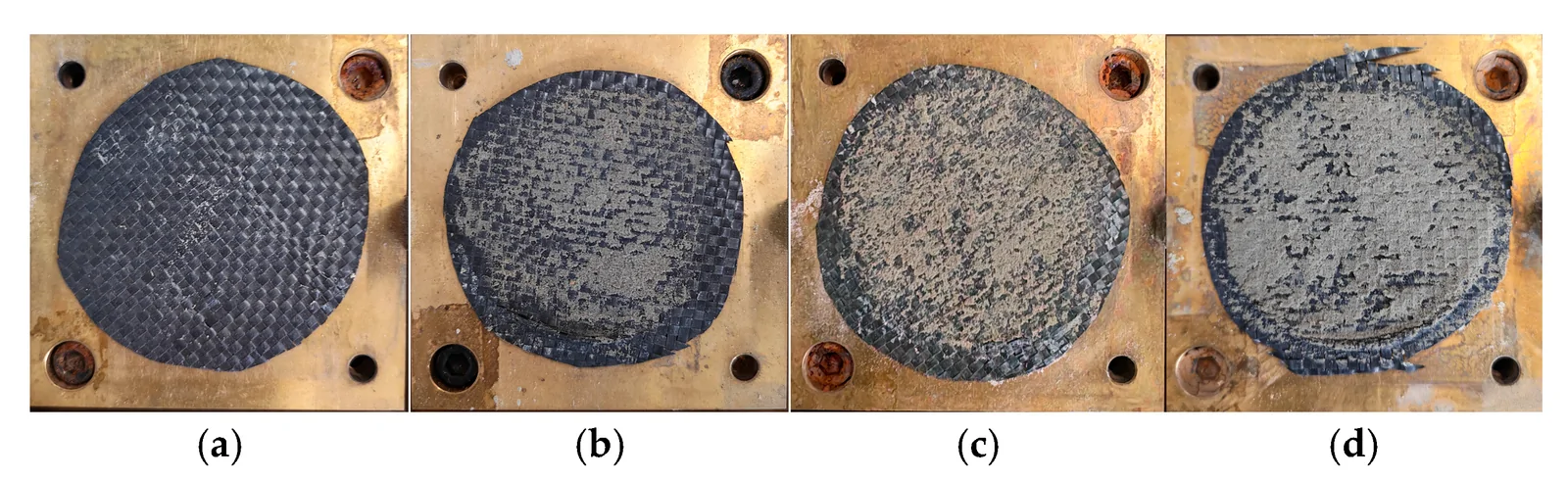
Shear surface morphologies after shear tests under different interlayer tailings contents: (**a**) 0 g/cm^2^; (**b**) 0.0125 g/cm^2^; (**c**) 0.025 g/cm^2^; (**d**) 0.25 g/cm^2^.

To further investigate the interaction mechanisms of the geotextile–tailings–geotextile interface under different interlayer tailings contents, after the shear tests, the failure patterns of various shear surfaces were observed under a normal stress of 200 kPa and a tailings water content of 20%. The pictures of the shear surface after the test are shown in Figure 11.

As shown in Figure 11a, after shear failure of the geotextile interface without interlayer tailings, very few tailings particles passed through the gaps between the warp and weft threads of the geotextile and remained on its surface. Observation of Figure 11b–d reveals that after shear failure, a certain number of the tailings adhered to the surface of the geotextile, while a number of tailings particles became embedded in the gaps between the warp and weft threads. The shear strength at the interface stemmed from the adhesion and interlocking effects between the geotextile and these tailings particles. When the tailings content per unit area of geotextile was 0.0125 g/cm^2^, the tailings on the interface were almost entirely embedded within the grid formed by the warp and weft threads of geotextile. However, many grid cells remained free of tailings. When the tailings content per unit area of geotextile increased to 0.0250 g/cm^2^, the tailings essentially filled the entire grid of the geotextile, and the adhesion between tailings particles and geotextile was further enhanced. Therefore, the shear strength of the interface was greater when the tailings content per unit area of geotextile was 0.0250 g/cm^2^ than when it was 0.0125 g/cm^2^. As the tailings content between geotextile layers continued to increase, the surface of geotextile became fully covered with tailings. Consequently, the contact area between tailings particles and geotextile ceased to expand, and the shear interface at the geotextile–tailings–geotextile junction gradually flattened, becoming more similar to the shear interface of the tailings itself. Therefore, the shear strength of the interface was lowest when the tailings content per unit area of geotextile was 0.0500 g/cm^2^.

Although the experimental results provide useful insights into the shear behavior of the geotextile–tailings–geotextile composite interface, the applicability of these findings should be considered within the specific material and testing conditions investigated in this study. The geotextile and tailings used in the tests were obtained from a specific engineering project. Different geotextile types, as well as variations in tailings mineralogy and particle size distribution, may alter the degree of particle–geotextile interlocking and consequently affect the interface shear strength. Therefore, the quantitative results, particularly the optimum interlayer tailings content of 0.0250 g/cm^2^, should not be directly generalized to other geotextile–tailings systems.

In addition, the present direct shear tests were conducted under normal stresses ranging from 50 to 200 kPa and under monotonic loading conditions. The applicability of the obtained interface parameters under higher normal stresses or long-term cyclic loading therefore requires further verification. Furthermore, long-term environmental exposure, including wetting–drying cycles, ultraviolet radiation, and temperature variations, may alter the surface properties and mechanical behavior of geotextiles and consequently affect their interfacial shear performance. These effects were not considered in the present study.

Further studies involving different geotextile types, tailings mineralogy and particle size distributions, wider normal-stress ranges, cyclic loading, and environmental aging are needed to establish more general interface shear-strength criteria for long-term engineering applications.

## 4. Stability Analysis of Tailings Dam Built with Geotextile Bags Based on Discontinuous–Continuous Coupling Method

### 4.1. DDA-FEM Coupling Method

Each sub-dam in a tailings dam built with geotextile bags consists of multiple geotextile bodies; a fully consolidated geotextile body can be regarded as a monolithic material, whereas the connections between geotextile bodies are not tight. The interactions between geotextile bodies, as well as between the sub-dam and tailings, constitute typical discontinuous problems. The Discontinuous Deformation Analysis (DDA) method [30] possesses distinct advantages in simulating block deformation and interface slippage, thus being suitable for analyzing various failure modes of slopes [31]. The DDA method was employed to simulate the deformation and displacement of geotextile bodies. Since the tailings in the reservoirs can be regarded as a continuous medium, a coupled DDA and finite element method (FEM) approach was adopted to analyze the stability of a tailings dam built with geotextile bags. The non-continuous model was applied to the geotextile dam section, while the continuous medium model was applied to the tailings in the reservoirs. Data transfer between the DDA and FEM computational domains was achieved through a transition layer.

**Figure 12:**
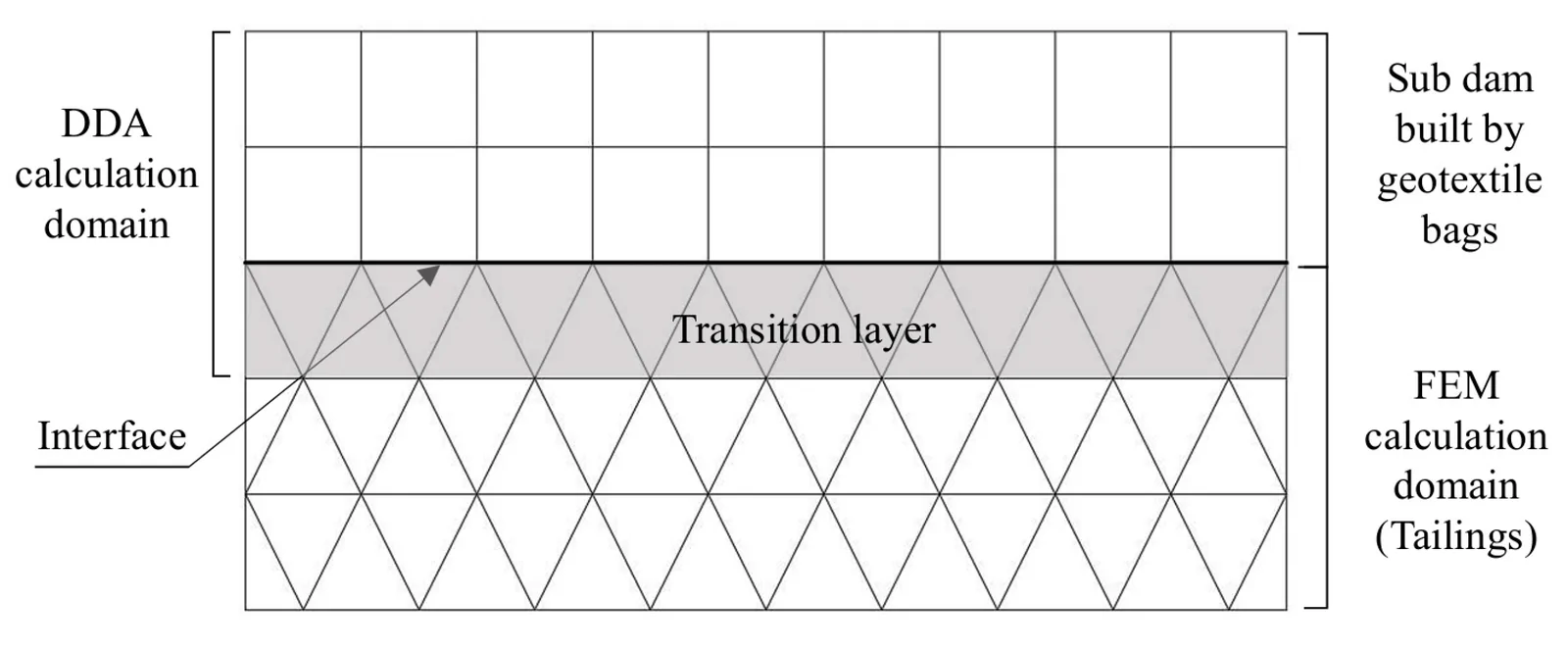
General calculation model of the DDA-FEM coupling method.

The DDA-FEM calculation model is shown in Figure 12. The outermost finite elements at the interface between the continuous and discontinuous media serve as the transition layer. The elements in the transition layer represent the continuous medium and are included in the calculations for the discontinuous medium. The results of these calculations are transferred between the two media through the transition layer.

**Figure 13:**
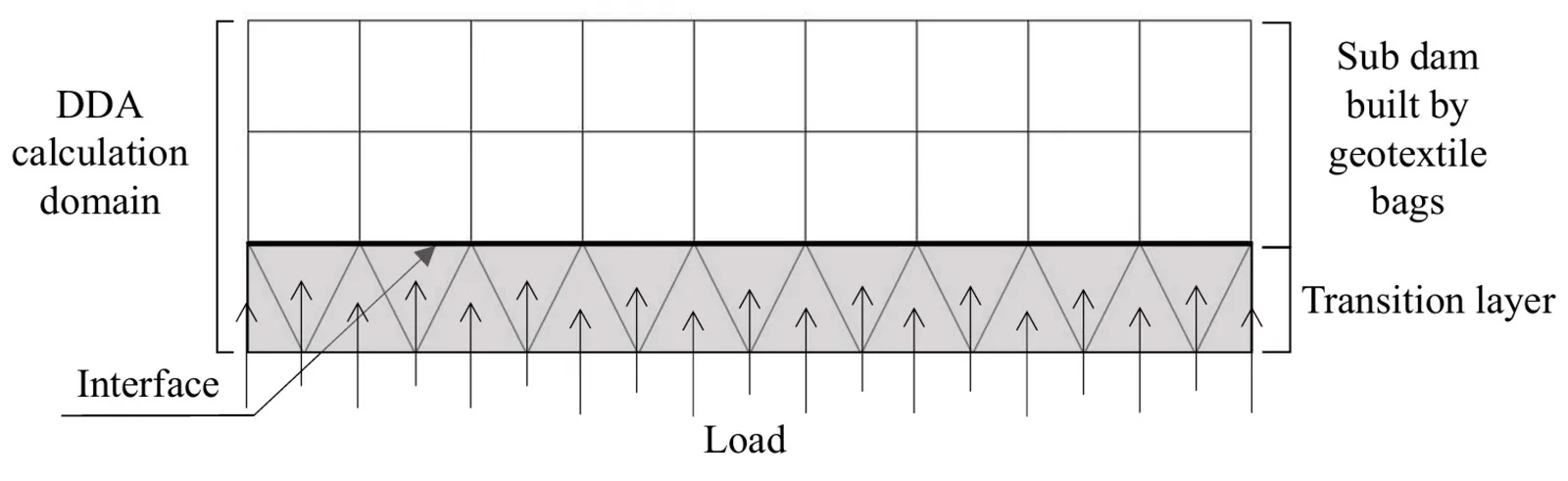
Transfer process of the load.

**Figure 14:**
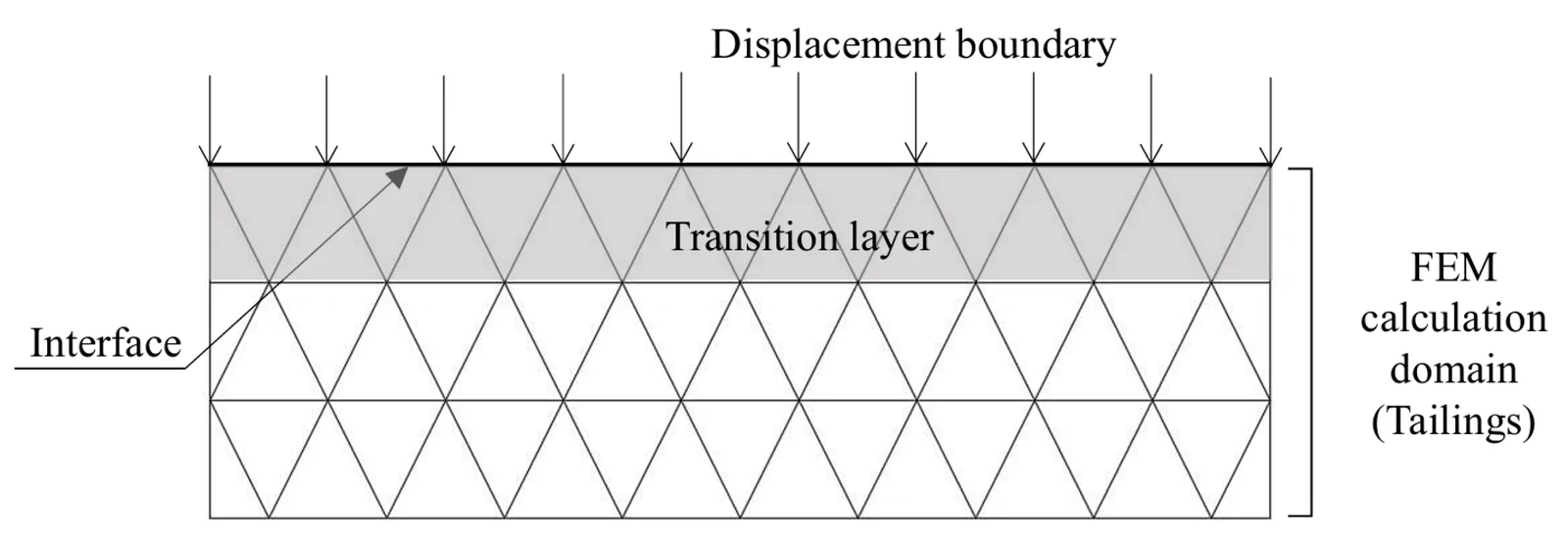
Transfer process of displacement.

The procedure for DDA-FEM coupling analysis is as follows:

(1) The initial displacement boundary at the nodes on the interface is set to zero, and the finite element analysis is executed. Then the equivalent node forces at the interface are obtained.
(2) Nodes on the interface generally correspond to multiple elements in the transition layer. The equivalent node forces are averaged over the number of elements corresponding to each node and then applied in the opposite direction to the corresponding transition layer elements through loading points, as shown in Figure 13. N steps of DDA calculation are performed to obtain the displacement increments at the outer edge points of the transition layer.
(3) If a node corresponds to multiple edge points of the transition layer, the displacement increments at each edge point are averaged, and then they are summed and applied as a displacement boundary to the corresponding finite element node, as shown in Figure 14. A finite element analysis was performed to determine the equivalent node forces at the interface.
(4) Steps (2) and (3) are repeated until the DDA block displacement increments satisfy the iteration criteria.

**Figure 15:**
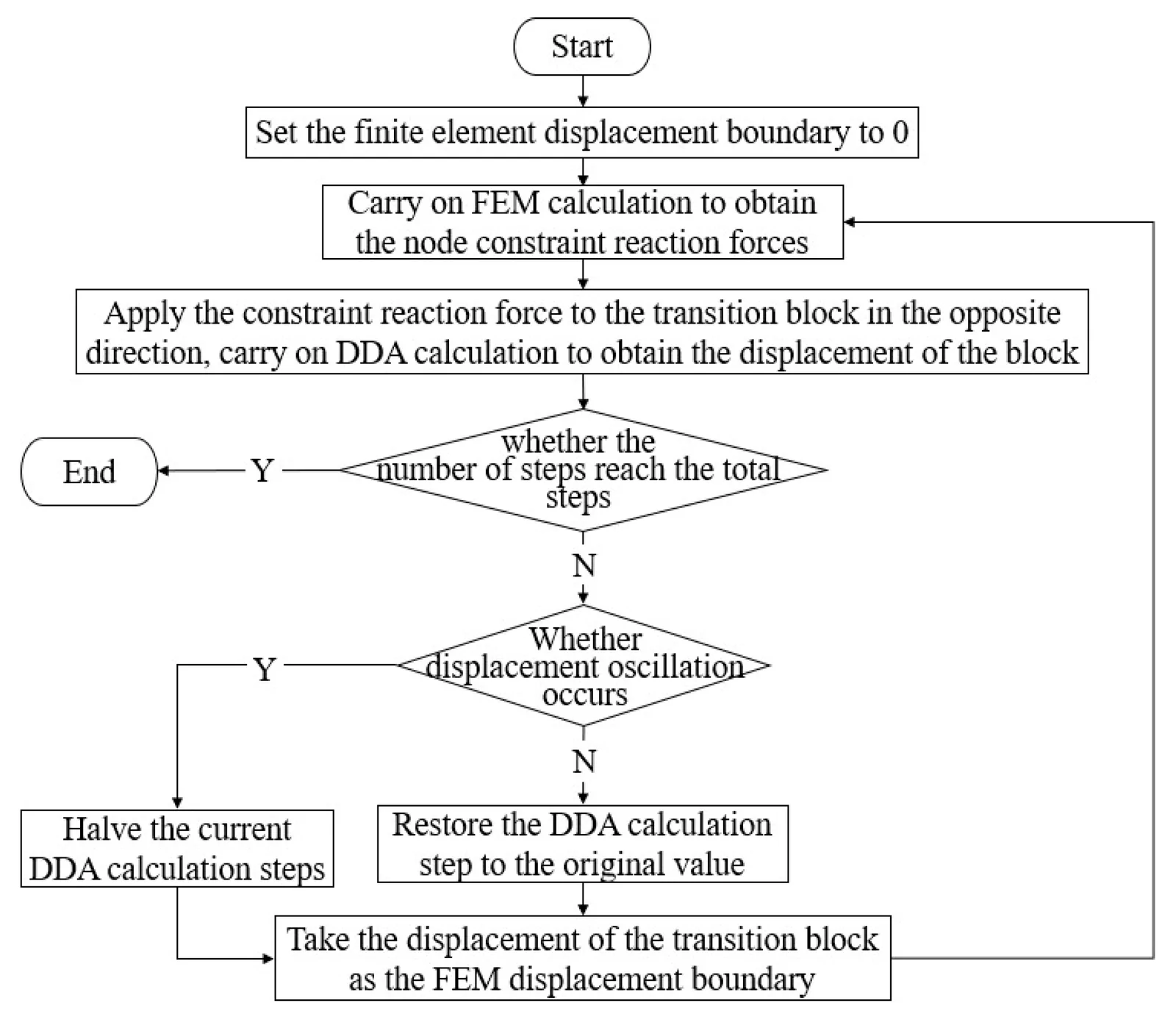
Flowchart of DDA-FEM coupling algorithm.

The algorithm flowchart for the DDA-FEM coupling method is shown in Figure 15.

### 4.2. Validation of the DDA-FEM Coupling Method

To verify the ability of the DDA-FEM coupled method to simulate friction, contact, and sliding behavior between discontinuous and continuous media, a slope-slider calculation example was established. This example simulates the process of an isolated block sliding down a slope under its own weight and verifies the accuracy of the coupling method in determining the block’s initiation of sliding by comparing the critical angle of friction obtained from numerical calculations with the theoretical analytical solution.

**Figure 16:**
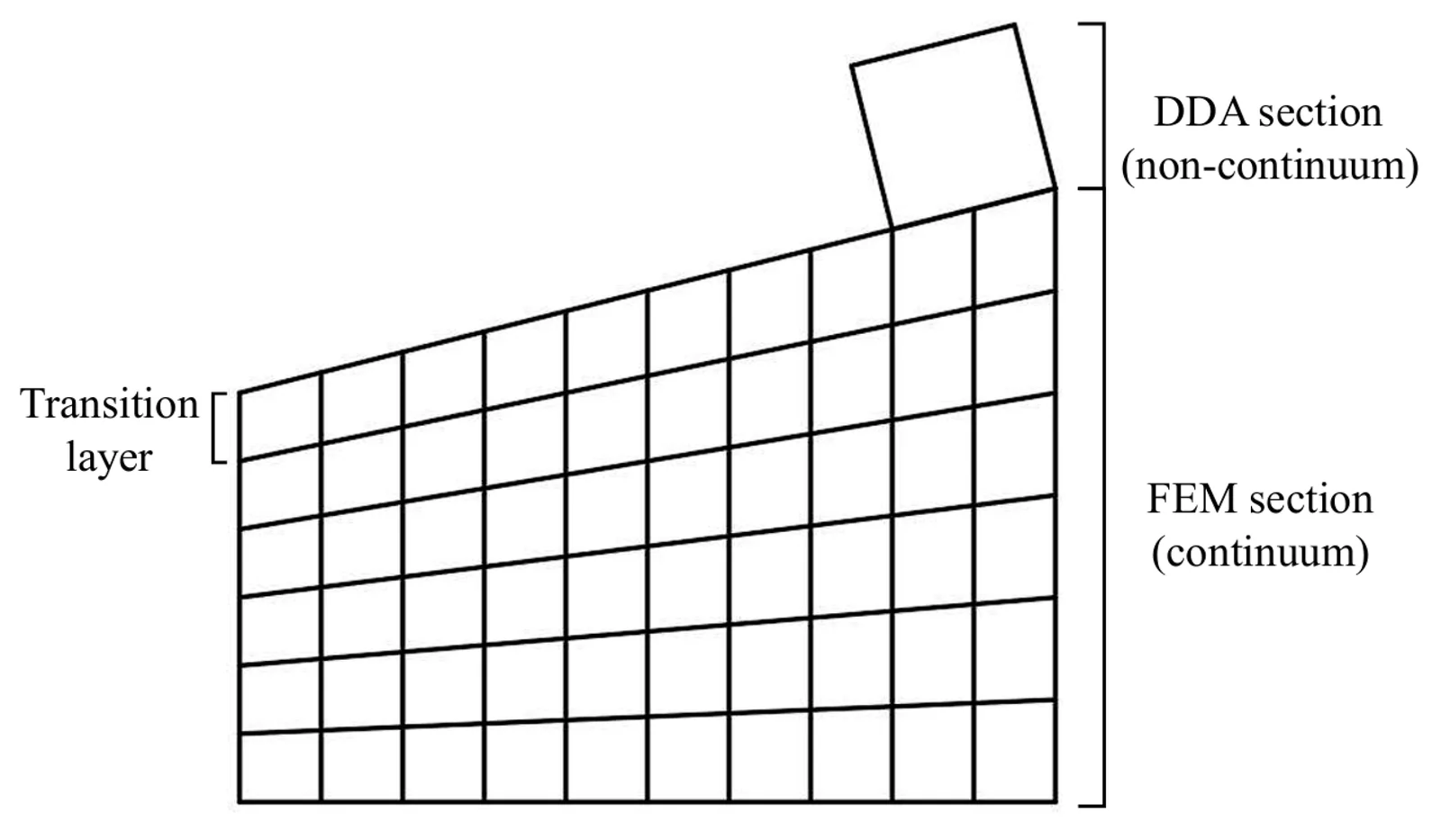
Example 1 calculation model.

In example 1, the slope is defined as a finite element model, with a height of 2 m on the left and 3 m on the right, and a base length of 4 m. The bottom nodes are fixed, and the model and its mesh are shown in Figure 16. The elastic modulus of the finite element material is 0.01 MPa, and the Poisson’s ratio is 0.30; its self-weight is not considered. The DDA model is an independent square block located at the top of the slope, with a density of 2 kN/m^3^, a modulus of elasticity of 0.01 MPa, and a Poisson’s ratio of 0.25. The transition layer consists of a single layer of elements in the finite element model adjacent to the DDA-FEM interface.

In the calculation, the cohesion and tensile strength at the interface between the slope and the sliding block were not considered; only interfacial friction was taken into account. The initial friction angle of the interface between the slope and the sliding block is set as 15°; the friction angle is then gradually reduced in 0.1° increments. When the friction angle decreases to 14.0°, the DDA block begins to slide, whereas it remains stable at 14.1°. Therefore, the critical friction angle obtained from the numerical calculations is 14.1°. According to the theoretical limit equilibrium conditions corresponding to the slope angle, the theoretical critical friction angle is 14.03°; the relative error between the numerical results and the theoretical analytical solution is approximately 0.5%.

The numerical result agrees well with the theoretical analytical solution, indicating that the DDA-FEM coupling method can reliably simulate the frictional contact behavior between discontinuous blocks and the continuous medium, and accurately predict the slip-off state of the block under friction-controlled conditions.

To further validate the contact and force transmission behavior between the discontinuous and continuous media during the DDA-FEM coupling process, a numerical example of multiple DDA blocks freely falling and coming into contact with the FEM continuous medium was established. This example focuses on examining the variation in the reaction forces at boundaries on the FEM side as the DDA block transitions from free movement and contact with the continuous medium to its final state of static equilibrium.

**Figure 17:**
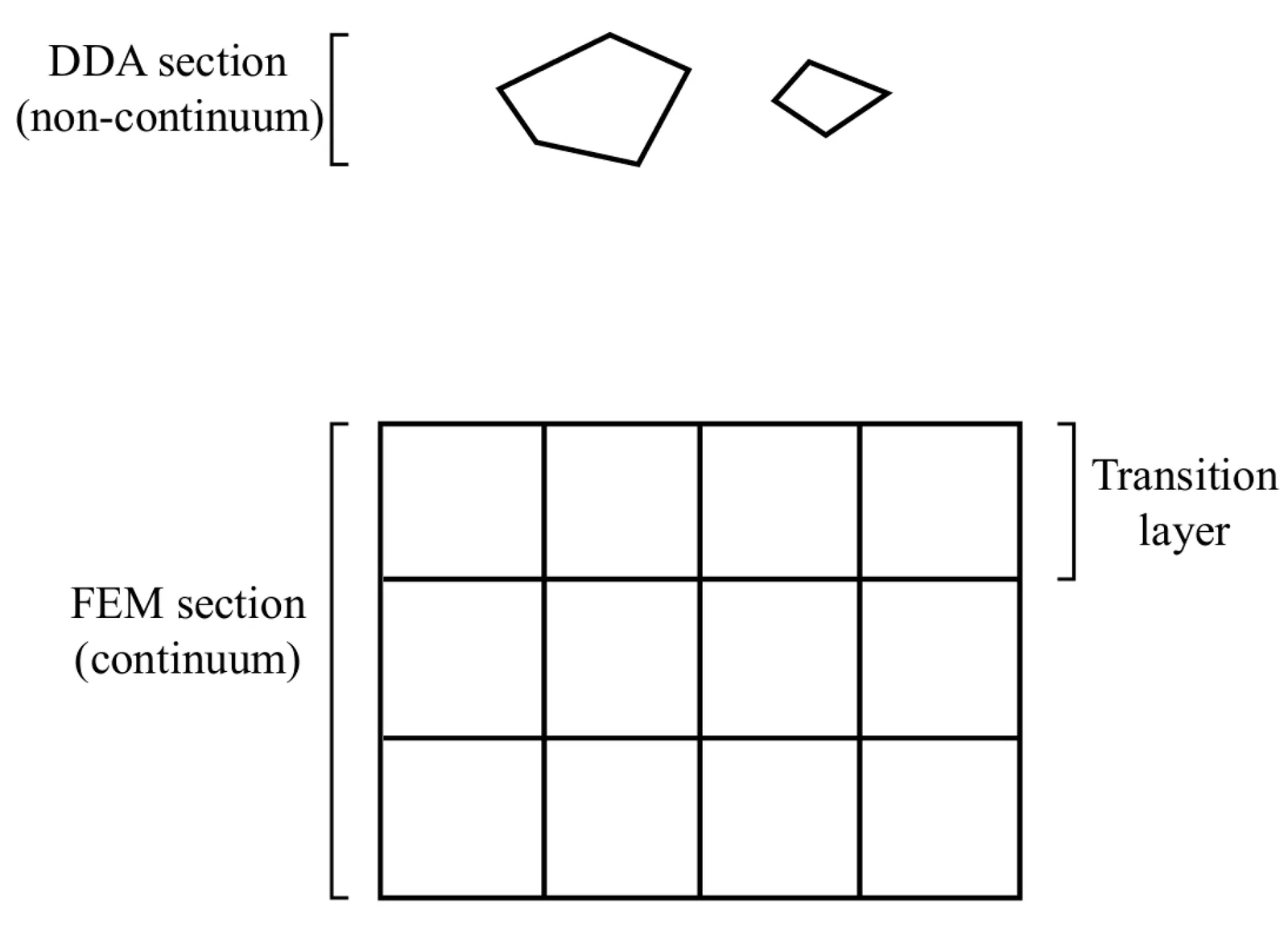
Example 2 calculation model.

In example 2, the finite element model consists of a platform 3 m high and 4 m wide. The DDA model consists of two separate blocks with different dimensions positioned above the platform. The self-weight, modulus of elasticity, and Poisson’s ratio of all materials are the same as in the previous example. The coupling model and its mesh are shown in Figure 17; the transition layer consists of the top four elements of the finite element model.

**Figure 18:**
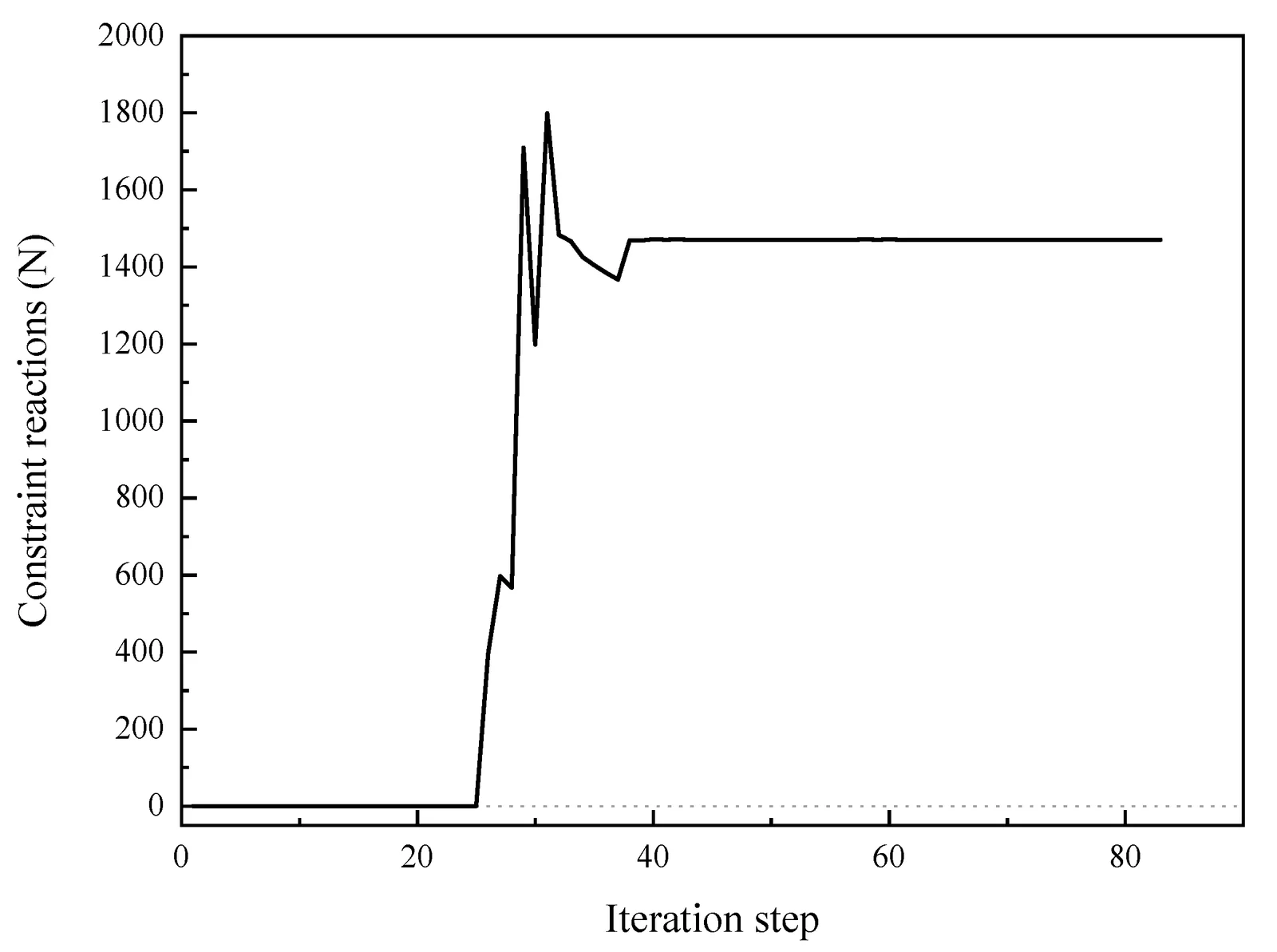
The curve of constraint reactions with iteration steps.

In the DDA-FEM coupling process, the interface constraint reactions obtained from the finite element analysis serve as external loads transferred from the FEM domain to the DDA domain; their computational accuracy directly affects the results of the coupling analysis. Therefore, this example simulates the entire process of two DDA blocks—from their initial free fall and contact with the FEM continuous medium, through movement adjustments, to the final attainment of static equilibrium—and records the total force of all node constraint reactions at the FEM-DDA interface for each iteration step, as shown in Figure 18.

As shown in Figure 18, as the DDA blocks come into contact with the FEM continuous medium, the blocks undergo processes such as collision and rotation, causing the interface constraint reaction to change dynamically; as the blocks gradually reach static equilibrium, the constraint reaction tends to stabilize. Ultimately, the resultant force of the interface constraint reaction on the FEM side stabilizes at 1.4711 kN, while the total self-weight of the two DDA blocks is 1.4778 kN; the relative error between the two is less than 0.5%.

The results indicate that, during the process by which the blocks transition from a non-contact state to a contact state and ultimately reach static equilibrium, the DDA-FEM coupling method is effective in transmitting the interaction forces between the DDA and FEM domains.

Based on the two numerical examples above, the DDA-FEM coupling method demonstrates high computational accuracy in both friction-controlled sliding problems and force transfer between discontinuous and continuous media. These results support the applicability of the DDA-FEM coupling method to the discontinuous–continuous interaction involved in the stability analysis of tailings dams built with geotextile bags.

### 4.3. The Influence of Tailings Content Between Geotextile Layers on the Stability of Tailings Dam

To evaluate the effect of tailings content between geotextile layers on the stability of the tailings dam, four groups of interlayer shear strength parameters were set based on the test results in this paper, and the safety factor of the tailings dam was calculated under various conditions of tailings content between geotextile layers when the tailings water content was 20%. Accounting for the nonlinear behavior of tailings, nonlinear finite element analysis was employed to characterize the deformation and displacement of tailings in the reservoirs. The stress and strain distribution of tailings under external loads and boundary conditions were analyzed by adopting an ideal elasto-plastic model and the Mohr–Coulomb yield criterion, and the solution was calculated using the margin iteration method. In the stability analysis of the tailings dam built with geotextile bags, the safety factor was calculated through the strength reduction method.

**Figure 19:**
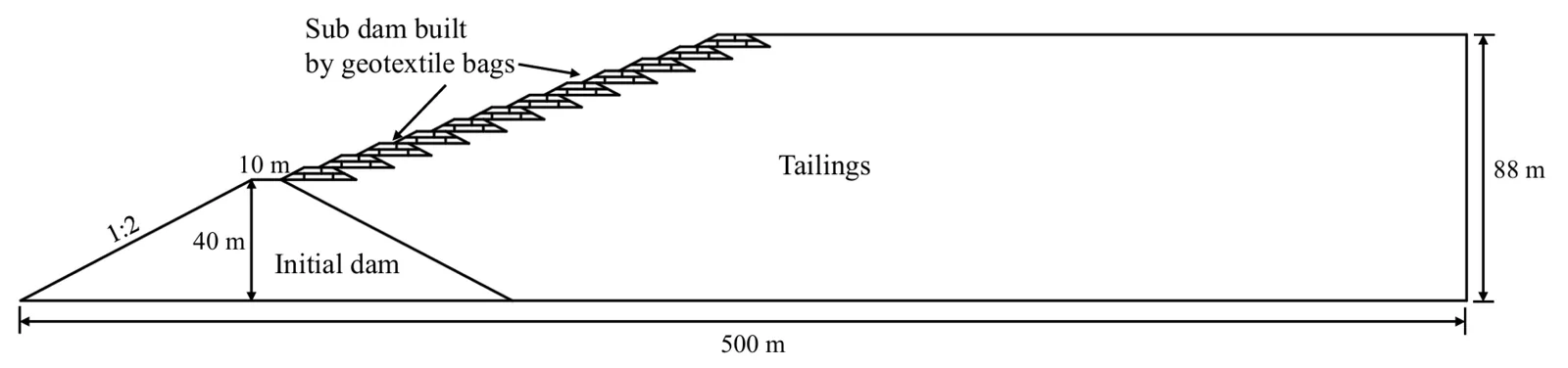
Calculation model of the tailings dam built with geotextile bags.

The computational model for the tailings dam is shown in Figure 19. The total dam height was 88 m, and the base width was 500 m. The initial dam had a height of 40 m and a crest width of 10 m, with upstream and downstream slopes both 1:2. The tailings dam was loaded with 12 levels of sub-dams built by geotextile bags. The overall slope ratio of the geotextile dam was 1:3. Each sub-dam was 4 m high, and the slope ratio on both the inner and outer sides was 1:2. The bottom boundary of the model is fixed; the left side corresponds to the initial dam, which is treated as a stable foundation in this analysis. It is assumed to remain stationary during the calculation, so no displacement occurs at the left boundary of the initial dam; the right boundary restricts normal displacement. The gravity of the tailings was 18.8 kN/m^3^, and the internal friction angle was 16°. The cohesion is 8 kPa, and the elastic modulus is 4985 kPa, with the Poisson’s ratio of 0.4. The gravity of the transition layer was set at 0.001 kN/m^3^, with the elastic modulus and Poisson’s ratio the same as those of the tailings. The internal friction angle at the interface between the geotextile and the tailings was set at 20.41°, and the cohesion was 8.66 kPa.

The simulated working conditions and corresponding safety factors of the tailings dam built with geotextile bags are shown in Table 4. From Table 4, as the tailings content between geotextile layers increased, the safety factor of the tailings dam first increased and then decreased, consistent with the law in the friction angle of the geotextile interface. When the tailings content between geotextile layers was 0.050 g/cm^2^, the safety factor of the tailings dam built with geotextile was 2.0, which was lower than that of the condition without tailings between geotextile layers.

**Figure 20:**
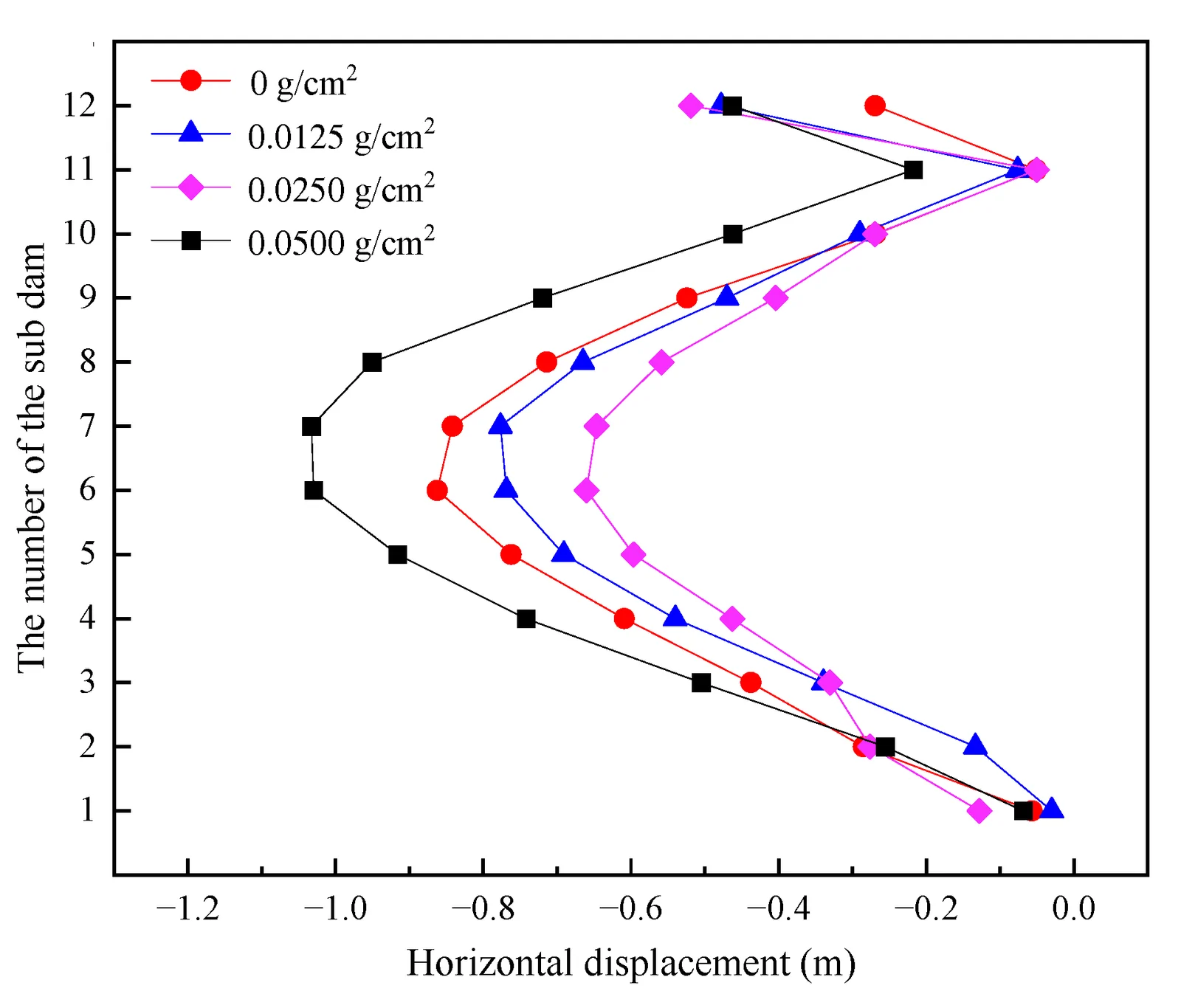
Horizontal displacement of the sub-dams under different tailings contents between geotextile layers.

When the calculation reached 1500 steps, the plastic zone of the tailings dam had extended from the bottom to the top of the slope under all four working conditions. Figure 20 shows the horizontal displacement of each level of the sub-dams built by geotextile bags at 1500 iteration steps. At this iteration step, as the tailings content between geotextile layers increased, the horizontal displacements of sub-dams first increased and then decreased. The distribution patterns of displacement across the sub-dams remained consistent, with peak horizontal displacements consistently occurring at the Level 6 and Level 7 sub-dams. Compared to conditions without tailings between geotextile layers, when the tailings content per unit area of geotextile was 0.025 g/cm^2^, the horizontal displacements of the Level 6 and Level 7 sub-dams decreased by 23.4% and 23.2%, respectively. When the tailings content per unit area of geotextile increased to 0.050 g/cm^2^, the horizontal displacement of the Level 6 and Level 7 sub-dams increased by 19.4% and 22.7%, respectively, compared to conditions without tailings between geotextile layers. This indicates that an appropriate amount of tailings between geotextile layers is beneficial to the safety of the tailings dam. However, when there is an excessive amount of tailings between geotextile layers, the shear strength of the geotextile interface will decrease significantly. This led to a remarkable increase in the horizontal displacement of the sub-dams built by geotextile across all levels, thus reducing the overall slope stability of the tailings dam built with geotextile bags. During the construction of a tailings dam built with geotextile bags, complete removal of residual tailings from the geotextile surface is not necessary. Instead, excessive accumulation of tailings should be avoided, and the residual tailings content should be appropriately controlled to maintain adequate interface shear resistance.

## 5. Conclusions

The influence of tailings content between geotextile layers on the shear properties at the geotextile–tailings–geotextile interface and the stability of the tailings dam built with geotextile bags was investigated through direct shear tests and DDA-FEM-coupled numerical analysis. The main conclusions are as follows:

(1) The combined effects of normal stress, tailings water content, and interlayer tailings content influenced the shear strength at the geotextile interface. The peak shear stress at the interface increased linearly with normal stress, and the interface shear strength followed the Mohr–Coulomb criterion. Under equal working conditions, the peak shear stress and friction angle at the interface with a 20% water content were generally higher than those at the interface with a 30% water content. The tailings content between geotextile layers significantly affected the shear performance of the interface. Under the material properties and experimental conditions investigated in this study, both the peak shear stress and the angle of friction initially increased and then decreased as the sand content increased, reaching a maximum when the tailings content per unit area was 0.0250 g/cm^2^. Cohesion followed a trend of first decreasing and then increasing as the tailings content increased.
(2) Changes in the shear strength of the geotextile interface were primarily controlled by particle interlocking at the interface. An appropriate amount of tailings particles can fully embed into the voids of the geotextile, forming a stable particle–geotextile mechanical interlock structure that increases interface roughness and enhances the particle interlocking effect, thereby significantly improving the interface’s shear resistance. When the tailings content between geotextile layers was overly high, a continuous, loose layer of tailings formed on the geotextile surface, causing the shear plane to gradually shift from the geotextile–tailings–geotextile interface to within the tailings itself. This intensified particle rolling and slippage, leading to a decrease in the interface friction angle and a reduction in shear strength.
(3) The tailings content between geotextile layers directly affected the entire tailings dam’s stability. As the tailings content between geotextile layers increased, the safety factor of the tailings dam first increased and then decreased. When the tailings content per unit area was 0.0250 g/cm^2^, the safety factor reached its maximum of 2.40, which was approximately 14.3% higher than under conditions without tailings. When the tailings content increased to 0.0500 g/cm^2^, the safety factor of the tailings dam dropped to 2.00, which was lower than that under conditions without tailings. Under the material properties and experimental conditions investigated in this study, controlling the interlayer tailings content can contribute to improving the overall stability of tailings dams built with geotextile bags. However, the identified value of 0.0250 g/cm^2^ should be regarded as a condition-dependent optimum rather than a universal threshold, and its applicability under different tailings gradations, densities, moisture contents, and geotextile conditions requires further investigation.

## Figures and Tables

**Table 1:** Physical and mechanical parameters of tailings in the geotextile bags.

Specific Gravity	Natural Density (g/cm^3^)	Natural Water Content (%)	Natural Void Ratio	Liquid Limit (%)	Plastic Limit (%)	Friction Angle (°)	Cohesion (kPa)
2.76	1.72	12	0.82	28	21	16.8	10.5

**Table 2:** Basic mechanical parameters of geotextile.

Mass (g/m^2^)	Tensile Strength (kN/m)		Elongation at Break (%)		Trapezoidal Tearing Strength (N)	
	Warp Direction	Weft Direction	Warp Direction	Weft Direction	Warp Direction	Weft Direction
160	30	22	28	28	>400	>400

**Table 3:** Shear test scheme for geotextile interface.

Group	Water Content of Tailings (%)	Interface Type	Tailings Content per Unit Area of Geotextile (g/cm^2^)	Normal Stress (kPa)
1	20	Geotextile	0	50, 100, 150, 200
2		Geotextile–Tailings–Geotextile	0.0125	
3			0.0250	
4			0.0500	
5	30	Geotextile	0	50, 100, 150, 200
6		Geotextile–Tailings–Geotextile	0.0125	
7			0.0250	
8			0.0500	

**Table 4:** The safety factor of the tailings dam under different tailings contents between geotextile layers.

	Tailings Content (g/cm^2^)	Friction Angle (°)	Cohesion (kPa)	Safety Factor of Tailings Dam
1	0	19.44	5.94	2.10
2	0.0125	20.46	5.81	2.20
3	0.0250	24.18	4.32	2.40
4	0.0500	16.75	10.30	2.00

## Data Availability

The original contributions presented in this study are included in the article. Further inquiries can be directed to the corresponding author.
